# Prostate brachytherapy with iodine-125 seeds: analysis of a single institutional cohort

**DOI:** 10.1590/S1677-5538.IBJU.2018.0142

**Published:** 2019-04-01

**Authors:** Elton Trigo Teixeira Leite, João Luis Fernandes da Silva, Eduardo Capelletti, Cecilia Maria Kalil Haddad, Gustavo Nader Marta

**Affiliations:** 1 Departamento de Radioterapia, Hospital Sirio-Libanês, São Paulo, SP, Brasil;; 2 Serviço de Radioterapia - Departamento de Oncologia da Universidade de São Paulo, Instituto do Câncer do Estado de São Paulo (ICESP), São Paulo, SP, Brasil

**Keywords:** Brachytherapy, Iodine-125 [Supplementary Concept], Prostate, Radiotherapy

## Abstract

**Objectives:**

Brachytherapy (BT) with iodine-125 seeds placement is a consolidated treatment for prostate cancer. The objective of this study was to assess the clinical outcomes in patients with prostate cancer who underwent low-dose-rate (LDR) -BT alone in a single Brazilian institution.

**Materials and Methods:**

Patients treated with iodine-125 BT were retrospectively assessed after at least 5 years of follow-up. Patients who received combination therapy (External beam radiation therapy-EBRT and BT) and salvage BT were not included.

**Results:**

406 men were included in the study (65.5% low-risk, 30% intermediate-risk, and 4.5% high-risk patients). After a median follow-up of 87.5 months, 61 (15.0%) patients developed biochemical recurrence. The actuarial biochemical failure-free survival (BFFS) at 5 and 10 years were 90.6% and 82.2%, respectively. A PSA nadir ≥ 1 ng / mL was associated with a higher risk of biochemical failure (HR = 5.81; 95% CI: 3.39 to 9.94; p ≤ 0.001). The actuarial metastasis-free survival (MFS) at 5 and 10 years were 98.3% and 94%, respectively. The actuarial overall survival (OS) at 5 and 10 years were 96.2% and 85.1%, respectively. Acute and late grade 2 and 3 gastrointestinal toxicities were observed in 5.6%, 0.5%, 4.6% and 0.5% of cases, respectively. For genitourinary the observed acute and late grade 2 and 3 toxicities rates were 57.3%, 3.6%, 28% and 3.1%, respectively. No grade 4 and 5 were observed.

**Conclusions:**

BT was effective as a definitive treatment modality for prostate cancer, and its endpoints and toxicities were comparable to those of the main series in the literature.

## INTRODUCTION

Prostate cancer is the second most common malignant neoplasm in men (excluding nonmelanoma skin cancer), with an estimated 1 million annual diagnoses worldwide (1). There are different treatment strategies for localized disease, which include radical prostatectomy, external radiotherapy, and brachytherapy (BT) (2). Many patients can be submitted to active surveillance and treated in a timely manner (2, 3).

Brachytherapy with iodine-125 seeds placement is a consolidated treatment and yields good results over a long clinical follow-up for patients with low and selected intermediate risk prostate cancer (4, 5). Large cohorts have demonstrated a rate of 86-87% and 79-80% of clinical control for low and intermediate risk respectively (6, 7). This modality remains the most conformal form of radiation dose delivery, allowing more effective dose escalation and good results when compared to external beam radiation therapy (EBRT) besides acceptable toxicity (2).

The objective of this study was to describe the biochemical failure-free survival (BFFS), metastasis-free survival (MFS), disease-specific survival (DSS), overall survival (OS), and treatment-related toxicities in patients with prostate cancer who underwent low-dose-rate (LDR) -BT alone in a single Brazilian institution.

## MATERIALS AND METHODS

Localized prostate cancer patients treated between March 2001 and November 2010 with BT were retrospectively assessed after a minimum of 5 years of follow-up. All patients clinically candidates for BT were submitted to digital rectal examination in position of lithotomy for assessment of the procedure feasibility technique. An ultrasonography was performed for pubic arch evaluation in patients with large prostatic gland and pubic arch interference was the only technical contraindication for the implant. BT was performed with real time intraoperative planning and iodine-125 seed implants guided by transrectal ultrasonography and radioscopy. The number of seeds implanted were variable according to prostate size and planning, and the range from 73 to 122 seeds per patient were used. For all patients, the prescribed dose was 144 Gy at 90% of prostate volume. After the seeds implant, the patients were submitted to post-implant dosimetry as suggested by American College of Radiology (ACR). The dose constraints used were V100 ≤ 1cc for the rectum and V150 < 50% for the urethra. Patients who received combination therapy (EBRT and BT), salvage BT and who were lost to follow-up were excluded.

Biochemical failure was defined according to the Phoenix criteria, a rise of 2 ng / mL above nadir. The BFFS, DSS, and OS were estimated using the Kaplan-Meier method. A log-rank test and multivariable Cox regression were used to evaluate the relationship of covariates with outcomes. The incidences of acute and late gastrointestinal and genitourinary toxicities and their respective confidence intervals (95% CI) were calculated using the National Cancer Institute Common Terminology Criteria for Adverse Events, version 4 (NCI CTCAE v4.0) scoring system (8).

The level of statistical significance adopted was p < 0.05. Statistical analyses were performed using the Stata™ statistical program (version 13.0) (9).

## RESULTS

Of the 616 patients treated with BT, 406 were included in the study. In total, 65.5% were low-risk, 30% were intermediate-risk, and 4.5% were high-risk patients. The patient's characteristics are described in Table-1.

**Table 1:** Demographic and clinical characteristics of prostate cancer patients undergoing brachytherapy (n = 406).

		n	%
**Age (years)**			
	< 60	113	27.8
	60 – 70	156	38.4
	> 70	137	33.7
**Color**			
	White	366	92.2
	Mixed	20	5.0
	Yellow	6	1.5
	Black	5	1.3
	No information	9	
**Comorbidities**			
	No	142	35.1
	Yes	263	64.9
	No information	1	
**Systemic hypertension**			
	No	271	67.1
	Yes	133	32.9
	No information	2	
**Cardiopathy**			
	No	342	84.2
	Yes	64	15.8
**Diabetes mellitus**			
	No	349	86.0
	Yes	57	14.0
**Hemorrhoids**			
	No	363	89.4
	Yes	43	10.6
**Gleason Score**			
	6	283	69.7
	7 (3 + 4)	64	15.8
	7 (4 + 3)	18	4.4
	8	13	3.2
	9	1	0.2
**Perineural invasion**			
	No	252	87.8
	Yes	35	12.2
	No information	119	
**Risk group** +			
	Low	266	65.5
	Intermediate	122	30
	High	18	4.4
**Total percentage of tumor (%)**			
	< 10	153	64
	10 – 19.9	69	24.7
	20 – 29.9	16	6.7
	30 – 39.9	7	2.9
	40 – 49.9	2	0.8
	≥ 50	2	0.8
	No information	167	
**First PSA after brachytherapy**			
	< 1 ng / mL	68	19
	≥ 1 ng / mL	289	81
	No information	49	
**PSA variation after brachytherapy**			
	PSA rising	42	11.8
	Decrease < 50%	119	33.3
	Decrease ≥ 50%	196	54.9
	No information	49	

+ D´Amico classification

**Figure 1:**
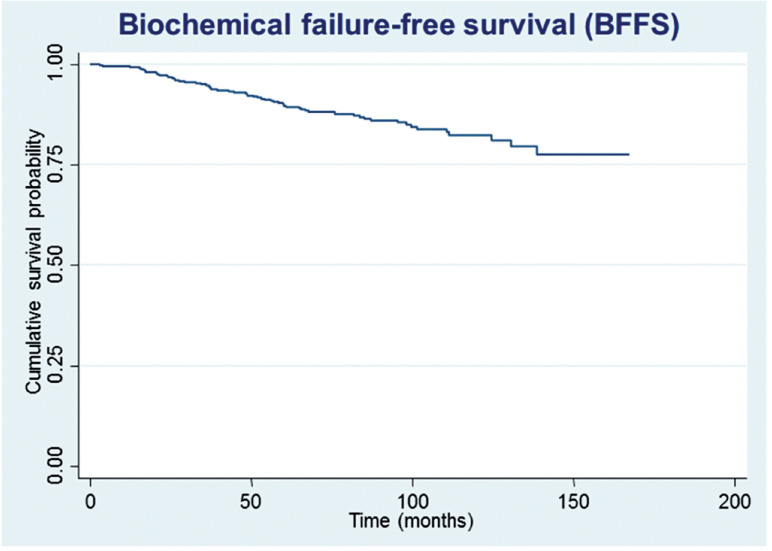
Estimates of biochemical failure-free survival in prostate cancer patients undergoing brachytherapy, obtained using the Kaplan-Meier method.

**Figure 2:**
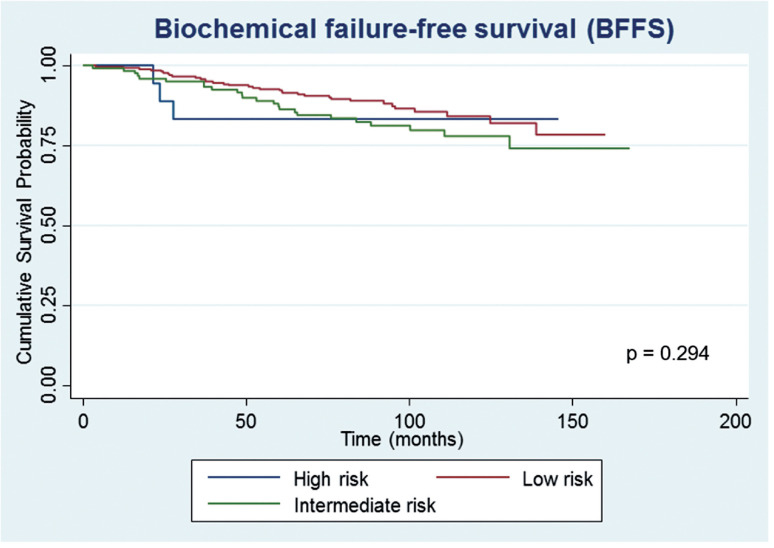
Estimates of biochemical failure-free survival in prostate cancer patients undergoing brachytherapy, obtained using the Kaplan-Meier method and according to the risk group.

After a median follow-up of 87.5 months, 61 (15.0%) patients developed biochemical recurrence. The actuarial BFFS at 5 and 10 years was 90.6% and 82.2%, respectively (Figure-1). There were no significant differences in the BFFS among the risk groups (p = 0.294) (Figure-2) and no significant associations between the BFFS and patient age, presence of comorbidities, perineural invasion, total tissue invasion, or Gleason score (Table-2). The mean PSA nadir was 0.53 ng / mL and a value ≥ 1 ng / mL was associated with a higher risk of recurrence (Figure-3A). Patients with a first PSA value (3 months after treatment) ≥ 1 ng / mL presented a higher risk of developing biochemical failure (Figure-3B). Analysis of patients whose PSA was first measured up to 60 days after BT showed that patients who had an increase in PSA after BT had a higher risk of biochemical failure than patients who presented a PSA reduction of more than 50.0% in relation to the PSA value collected before BT (HR = 2.26, 95% CI: 1.02 to 4.98); However, there was no significant difference in the risk of biochemical failure between patients who showed a reduction of less than 50.0% and those with reduction greater than 50.0%. The actuarial MFS at 5 and 10 years was 98.2% (95% CI: 96.3% to 99.1%) and 94% (95% CI: 89.9% to 96.5%), respectively. Seventeen (4.2%) patients had metastases (10 had bone metastases, 4 had visceral metastases, and 3 had lymph node metastases), 7 of whom died. Three patients (0.3%) died from prostate cancer and 4 died from cardiovascular causes during the follow-up. The actuarial OS at 5 and 10 years was 96.2% and 85.1%, respectively (Figure-4).

**Figure 3A:**
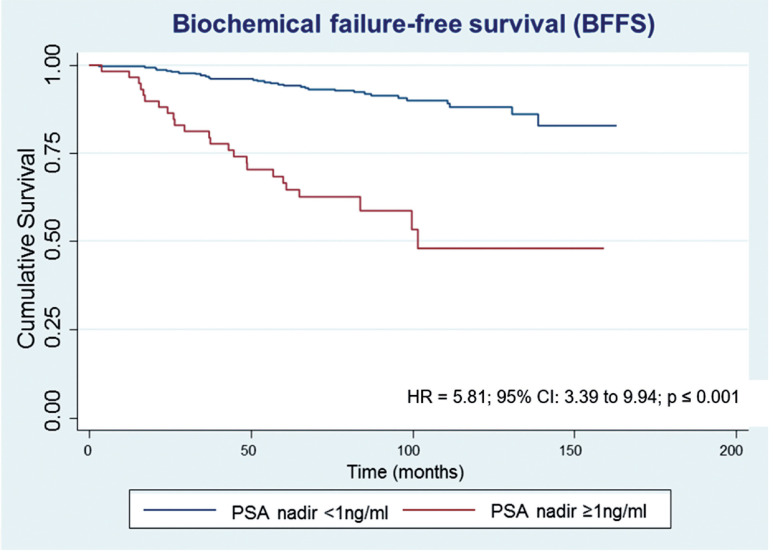
Estimates of biochemical failure-free survival in prostate cancer patients undergoing brachytherapy, obtained using the Kaplan-Meier method and according to the PSA nadir.

**Figure 3B:**
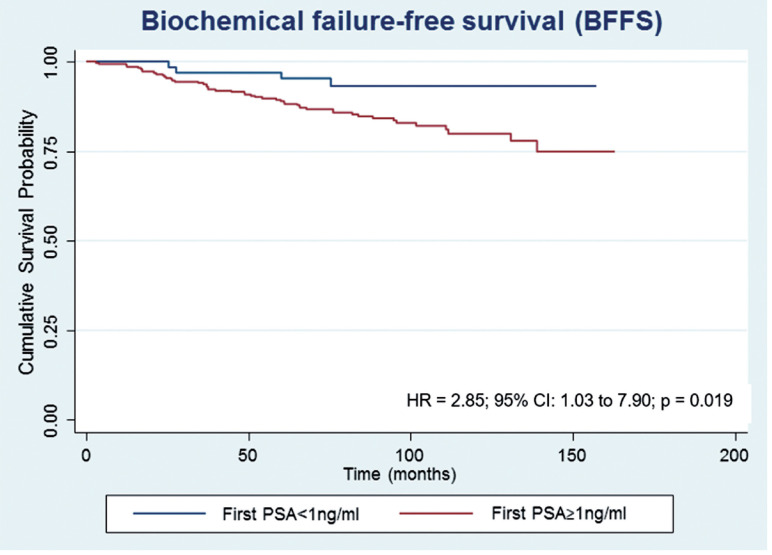
Estimates of biochemical failure-free survival in prostate cancer patients undergoing brachytherapy, obtained using the Kaplan-Meier method and according to the first PSA.

**Figure 4:**
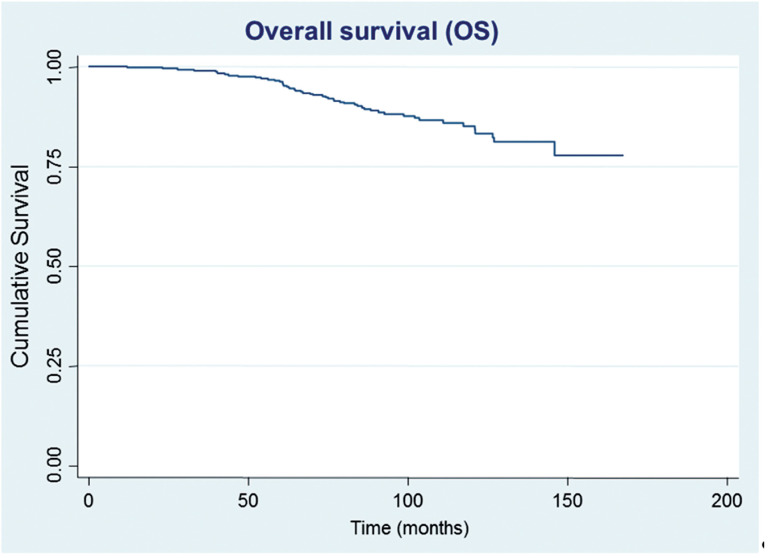
Estimates of overall survival in prostate cancer patients undergoing brachytherapy, obtained using the Kaplan-Meier method.

**Table 2:** Univariate analysis of biochemical failure-free survival according to patient characteristics and lesions of prostate cancer patients undergoing brachytherapy (n = 406).

		Biochemical control	Mean (months) (a)	SE	HR	95% CI	p
**Age (y)**							0.450
	< 70	87.2%	385.2	7.8	1.14	0.59-2.22	
	≥ 70	83.3%	361	11.9	1.51	0.76-3.01	
**Comorbidities** *							0.787
	No	84.5%	384.1	9.2	1		
	Yes	85.1%	366.8	9.4	1.07	0.64-1.81	
**Risk group** +							0.294
	Low	87.1%	329.3	41.6	1		
	Intermediate	80.0%	380.3	8.2	1.5	0.89-2.53	
	High	83.3%	379.2	7.2	1.55	0.48-5.04	
**Perineural Invasion**							0.133
	No	88.4%	395.1	8.4	1		
	Yes	76.5%	357.3	28.1	1.9	0.87-4.15	
**Total % of tumor** #							0.484
	< 10	89.5%	390.2	12	1		
	10-19.9	86.4%	297.2	22.9	1.27	0.55-2.98	
	≥ 20	90.4%	374.6	13.6	1.17	0.69-1.98	
**First PSA after treatment**							0.019
	< 1 ng / mL	94.0%	389.7	9.6	1		
	≥ 1 ng / mL	83.0%	370.4	8.6	2.85	1.03-7.90	
**PSA Nadir**							≤ 0.001
	< 1 ng / mL	90.3%	397.4	6.5	1		
	≥ 1 ng / mL	59.3%	294.9	19.9	5.81	3.39-9.94	
**Gleason score**							0.795
	6	85.5%	379.6	8.2	1		
	7 (3 + 4)	82.3%	351.5	14.4	1.09	0.56-2.12	
	7 (4 + 3)	86.4%	383.6	33.8	0.89	0.38-2.10	
	8 or 9	78.6%	321	6.7	1.83	0.57-5.91	

**(a)** = BFFS mean time; **SE** = Standard error;

* = Comorbidities: Systemic hypertension, cardiopathy, diabetes mellitus;

+ = D´Amico classification;

# = Proportion: Adenocarcinoma / normal prostate tissue;

**HR** = Hazard Ratio; **CI** = Confidence Interval; **p** = p Value of Log-Rank test

Acute grade ≥ 2 and grade ≥ 3 gastrointestinal toxicities were observed in 5.6% and 0.5% of the cases, respectively. Late grade ≥ 2 and grade ≥ 3 gastrointestinal toxicities were observed in 4.6% and 0.5% of the cases, respectively. There were no significant associations between age, diabetes mellitus, cardiopathy, hemorrhoids, neoadjuvant hormone therapy, prostate volume, and incidence of acute and late gastrointestinal toxicity.

The acute grade ≥ 2 and grade ≥ 3 genitourinary toxicity incidences were 57.3% and 3.6%, respectively. Late grade ≥ 2 and grade ≥ 3 symptoms were observed in 28% and 3.1% of patients, respectively. There were no significant associations between age, diabetes mellitus, cardiopathy, hemorrhoids, neoadjuvant hormone therapy, prostate volume, and incidence of genitourinary toxicity. Patients who had acute urinary retention had a greater prostate volume (median = 47.0 cm³, p_25_: 29.0 cm³; p_75_: 59.4 cm³) than those who did not have these features (median = 37.0 cm³; p_25_: 29.0 cm³; p_75_: 46.7 cm³) (p = 0.017).

Patients with a baseline International Prostate Symptom Score (IPSS) of greater than 10 were at an increased risk of acute genitourinary toxicity ≥ 2 (RR = 1.40, 95% CI: 1.16 to 1.69) (p = 0.004) and late genitourinary toxicity ≥ 2 (RR = 1.79; 95% CI: 1.26 to 2.56) (p = 0.005) (Tables 3 and 4).

**Table 3:** Characteristics associated with incidence of acute genitourinary toxicity ≥ 2 in prostate cancer patients undergoing brachytherapy (n = 393).

		Acute genitourinary toxicity ≥ 2				RR	95% CI		p
		No		Yes					
		(n = 168)		(n = 225)					
		n	%	n	%				
**Age group (years)**									0.924
	< 60	39	41.0	56	59.0	1			
	60 – 70	74	43.0	98	57.0	0.97	0.78	1.19	
	> 70	55	43.7	71	56.4	0.96	0.96	1.20	
**Diabetes mellitus**									0.305
	No	141	41.7	197	58.3	1			
	Yes	27	49.1	28	50.9	0.87	0.66	1.15	
**Cardiopathy**									0.173
	No	138	41.3	196	58.7	1			
	Yes	30	50.9	29	49.1	0.84	0.64	1.10	
**Neoadjuvant hormone therapy**									0.119
	No	135	41.0	194	59.0	1			
	Yes	33	51.6	31	48.4	0.82	0.63	1.07	
**IPSS**									0.004
	< 10	111	47.8	121	52.2	1			
	10 – 20	19	27.1	51	72.9	1.40	1.16	1.69	
	> 20	2	22.2	7	77.8	1.49	1.02	2.16	
**Risk group**									0.336
	Low	118	45.4	142	54.6	1			
	Intermediate	44	37.6	73	62.4	1.14	0.95	1.37	
	High	6	37.5	10	62.5	1.14	0.77	1.70	

**RR** = Relative risk; **IPSS** = International Prostate Symptom Score; **CI** = Confidence Interval; **p** = p Value of X^2^

**Table 4:** Characteristics associated with incidence of late genitourinary toxicity ≥ 2 in prostate cancer patients undergoing brachytherapy (n = 390).

		Late genitourinary toxicity ≥ 2				RR	95% CI		p
		No		Yes					
		(n = 281)		(n = 109)					
		n	%	n	%				
**Age group (years)**									0.231
	< 60	64	67.4	31	32.6	1			
	60 – 70	132	76.3	41	23.7	0.73	0.49	1.08	
	> 70	85	69.7	37	30.3	0.93	0.63	1.38	
**Diabetes mellitus**									0.281
	No	244	73.0	90	27.0	1			
	Yes	37	66.1	19	33.9	1.26	0.83	1.89	
**Cardiopathy**									0.483
	No	237	71.4	95	28.6	1			
	Yes	44	75.9	14	24.1	0.84	0.52	1.38	
**Neoadjuvant hormone therapy**									0.606
	No	238	72.6	90	27.4	1			
	Yes	43	69.4	19	30.6	1.12	0.74	1.69	
**IPSS**									0.005
	< 10	175	76.1	55	23.9	1			
	10 – 20	40	57.1	30	42.9	1.79	1.26	2.56	
	> 20	5	55.6	4	44.4	1.86	0.86	4.00	
**Risk group**									0.092
	Low	195	75.6	63	24.4	1			
	Intermediate	76	65.5	40	34.5	1.41	1.01	1.96	
	High	10	62.5	6	37.5	1.54	0.79	2.99	

**RR** = Relative risk; **IPSS** = International Prostate Symptom Score; **CI** = Confidence Interval; **p** = p Value of X^2^

## DISCUSSION

Prostate cancer is diagnosed at progressively earlier stages and the proportion of men with low-risk disease is increasing (10). The approach to low-risk prostate cancer involves active surveillance (11) or treatment with radical prostatectomy, external radiation therapy, or BT (2). Mostly, there are several published series comparing all treatment modalities, once data from prospective studies are not broadly available. Prostate BT has the same efficacy as other radical treatments on localized disease (6, 12-14).

Grimm et al. (7) published a series of 125 cases of prostate BT with iodine-125 seed implants. After 10 years of follow-up, a BFFS of 85.1% was achieved, and in low-risk patients, the rate was 87%. Kollmeier et al. (15) published an institutional experience with prostatic iodine-125 and palladium-103 implants after a minimum follow-up of 5 years. In total, 336 patients with localized disease were treated, and a BFFS of 77% was obtained. Disease-related factors, including the initial PSA level, Gleason score, and stage, were significant predictors of biochemical failure.

The present study demonstrated PSA control rates similar to those in the literature. The majority of patients treated in this cohort were low-risk and no statistically significant difference in outcomes among the risk groups were observed probably due to the poor representation of intermediate and high-risk patients.

Biochemical control rates demonstrated in this study can also be compared to the main series of dose escalation EBRT. The same institution reported outcomes of high dose EBRT with Intensity Modulation Radiation Therapy (IMRT) and the biochemical control rate was 86.4% after a median follow-up of 58 months. Five year BFFS was 91.7% for low risk patients (16).

A positive aspect of our cohort is that all patients were submitted to post-implant dosimetry. It is well know that an adequate prostate coverage with the prescription dose in the post-implant analysis is related to better BFFS (17). Pereira da Ponte Amadei et al. (18) published a retrospective data of first patients treated at the same hospital without post-implant dosimetry, and the BFFS rate was lower (80% of 5-year BFFS).

Variables related to clinical outcomes were also identified in this study. In our institutional experience, nPSA < 1 ng / mL was related to better chance of biochemical control with IMRT (19) and these findings were confirmed in the present BT cohort. Furthermore, patients who had this PSA value in the first measure after the procedure also have a better prognosis. Several studies have analyzed PSA dynamics after prostate cancer treatment with radiotherapy (20, 21). Ko et al. (22) associated nPSA < 0.5 ng / mL with a higher BFFS; in addition, those who achieved this value in the first 5 years after the procedure showed an even higher BFFS than those who achieved it after 5 years.

Data regarding the treatment-related toxicity were also collected in the present study. It is known that BT-related toxicity and its impact on quality of life are comparable to those of other treatment methods (5, 23, 24). There are several factors relating to greater morbidity in prostate BT, including IPSS, prior transurethral resection, large (> 60 cm^3^) or small (< 20 cm^3^) glands, acute prostatitis, and inflammatory bowel disease (25).

Our genitourinary toxicity results had comparable or lower rates in relation to the main series reporting these data (26, 27). It is known that prostate size is not necessarily a limiting factor in regard to undergoing treatment (28). In a series of 325 men treated with iodine-125 implants, Stone and Stock (29) did not notice a significant difference in urinary symptoms of patients with large and small prostates. However, our study demonstrated a relationship between acute urinary retention and prostate volume; therefore, patients with larger prostate volume have higher risk of acute urinary retention. Our gastrointestinal toxicity data were also quite encouraging and comparable to those of large prostate BT centers (30, 31). The BT-related toxicity may also be compared to EBRT. This analysis was performed in previous studies (14) and the conclusions are, in general, BT is associated with higher rates of acute urinary toxicities, mainly related to obstructive symptoms and urinary retention. Moreover, BT is related to lower rates of acute and late gastrointestinal toxicities. These aspects are consistent with our institutional data (16).

To the best of our knowledge, the present study represents the largest cohort with a long term follow-up of patients submitted to low dose BT in Brazil and Latin America and had showed satisfactory results. Although BT has been used less often in recent years in the US (32), it remains the most conformal form of radiation delivery as well as the optimal means for dose-escalation. Besides that, it is a quick, low-risk surgical procedure performed in a single day and a quick recovery for the patients. These characteristics are interesting for developing countries with poor installed capacity for radiation therapy institutions (33).

## CONCLUSIONS

BT with iodine-125 was effective at this institution as a definitive treatment modality for prostate cancer, and its endpoints and toxicities were comparable to those of the main series in the literature. Well-screened patients with low- and intermediate-risk prostate cancer should be offered this procedure as often as other therapeutic options, such as external radiotherapy and radical prostatectomy.

## Abbreviations

RP: = radical prostatectomy
BT: = brachytherapy
EBRT: = external beam radiation therapy
BFFS: = biochemical failure-free survival
MFS: = metastasis-free survival
DSS: = disease-specific survival
OS: = overall survival
LDR: = low-dose-rate
HR: = hazard ratio
p: = p value
NCI CTCAE: = National Cancer Institute Common Terminology Criteria for Adverse Events
PSA: = prostate-specific antigen
IPSS: = International Prostate Symptom Score
nPSA: = PSA nadir value

## References

[1] Fitzmaurice C, Allen C, Barber RM, Barregard L, Bhutta ZA, Brenner H (2017). Global, Regional, and National Cancer Incidence, Mortality, Years of Life Lost, Years Lived With Disability, and Disability-Adjusted Life-years for 32 Cancer Groups, 1990 to 2015: A Systematic Analysis for the Global Burden of Disease Study. JAMA Oncol.

[2] Kupelian PA, Potters L, Khuntia D, Ciezki JP, Reddy CA, Reuther AM (2004). Radical prostatectomy, external beam radiotherapy < 72Gy, external beam radiotherapy > or =72Gy, permanent seed implantation, or combined seeds/external beam radiotherapy for stage T1-T2 prostate cancer. Int J Radiat Oncol Biol Phys.

[3] Hamdy FC, Donovan JL, Lane JA, Mason M, Metcalfe C, Holding P (2016). 10-Year Outcomes after Monitoring, Surgery, or Radiotherapy for Localized Prostate Cancer. N Engl J Med.

[4] Critz FA, Benton JB, Shrake P, Merlin ML (2013). 25-Year disease-free survival rate after irradiation for prostate cancer calculated with the prostate specific antigen definition of recurrence used for radical prostatectomy. J Urol..

[5] Grimm P, Billiet I, Bostwick D, Dicker AP, Frank S, Immerzeel J (2012). Comparative analysis of prostate-specific antigen free survival outcomes for patients with low, intermediate and high risk prostate cancer treatment by radical therapy. Results from the Prostate Cancer Results Study Group. BJU Int.

[6] Sylvester JE, Grimm PD, Wong J, Galbreath RW, Merrick G, Blasko JC (2011). Fifteen-year biochemical relapse-free survival, cause-specific survival, and overall survival following I(125) prostate brachytherapy in clinically localized prostate cancer: Seattle experience. Int J Radiat Oncol Biol Phys.

[7] Grimm PD, Blasko JC, Sylvester JE, Meier RM, Cavanagh W (2001). 10-year biochemical (prostate-specific antigen) control of prostate cancer with (125)I brachytherapy. Int J Radiat Oncol Biol Phys.

[8] Ray A, Manjila S, Hdeib AM, Radhakrishnan A, Nock CJ, Cohen ML (2015). Extracranial metastasis of gliobastoma: Three illustrative cases and current review of the molecular pathology and management strategies. Mol Clin Oncol.

[9] Müller C, Holtschmidt J, Auer M, Heitzer E, Lamszus K, Schulte A (2014). Hematogenous dissemination of glioblastoma multiforme. Sci Transl Med.

[10] Cooperberg MR, Moul JW, Carroll PR (2005). The changing face of prostate cancer. J Clin Oncol.

[11] Tosoian JJ, Mamawala M, Epstein JI, Landis P, Wolf S, Trock BJ (2015). Intermediate and Longer-Term Outcomes From a Prospective Active-Surveillance Program for Favorable-Risk Prostate Cancer. J Clin Oncol.

[12] Morris WJ, Keyes M, Spadinger I, Kwan W, Liu M, McKenzie M (2013). Population-based 10-year oncologic outcomes after low-dose-rate brachytherapy for low-risk and intermediate-risk prostate cancer. Cancer.

[13] Taira AV, Merrick GS, Butler WM, Galbreath RW, Lief J, Adamovich E (2011). Long-term outcome for clinically localized prostate cancer treated with permanent interstitial brachytherapy. Int J Radiat Oncol Biol Phys.

[14] Sanda MG, Dunn RL, Michalski J, Sandler HM, Northouse L, Hembroff L (2008). Quality of life and satisfaction with outcome among prostate-cancer survivors. N Engl J Med.

[15] Kollmeier MA, Stock RG, Stone N (2003). Biochemical outcomes after prostate brachytherapy with 5-year minimal follow-up: importance of patient selection and implant quality. Int J Radiat Oncol Biol Phys.

[16] Gadia R, Leite ÉT, Gabrielli FG, Marta GN, Arruda FF, Abreu CV (2013). Outcomes of high-dose intensity-modulated radiotherapy alone with 1 cm planning target volume posterior margin for localized prostate cancer. Radiat Oncol.

[17] Zelefsky MJ, Kuban DA, Levy LB, Potters L, Beyer DC, Blasko JC (2007). Multi-institutional analysis of long-term outcome for stages T1-T2 prostate cancer treated with permanent seed implantation. Int J Radiat Oncol Biol Phys.

[18] Pereira da Ponte Amadei L, Fernandes Silva JL, Hanna SA, Haddad CM, Nesrallah AJ, Carvalho HA (2012). Biochemical control of prostate cancer with iodine-125 brachytherapy alone: experience from a single institution. Clin Transl Oncol.

[19] Gadia R, Teixeira Leite ET, Bierrenbach AL, Ynoe de Moraes F, Spratt DE, Arruda FF (2016). Long-term outcomes of dose-escalated intensity modulated radiation therapy alone without androgen deprivation therapy for patients with intermediate and high-risk prostate cancer. Adv Radiat Oncol.

[20] Iannuzzi CM, Stock RG, Stone NN (1999). PSA kinetics following I-125 radioactive seed implantation in the treatment of T1-T2 prostate cancer. Radiat Oncol Investig.

[21] Cavanaugh SX, Kupelian PA, Fuller CD, Reddy C, Bradshaw P, Pollock BH (2004). Early prostate-specific antigen (PSA) kinetics following prostate carcinoma radiotherapy: prognostic value of a time-and-PSA threshold model. Cancer.

[22] Ko EC, Stone NN, Stock RG (2012). PSA nadir of <0.5 ng/mL following brachytherapy for early-stage prostate adenocarcinoma is associated with freedom from prostate-specific antigen failure. Int J Radiat Oncol Biol Phys.

[23] D’Amico AV, Whittington R, Malkowicz SB, Schultz D, Blank K, Broderick GA (1998). Biochemical outcome after radical prostatectomy, external beam radiation therapy, or interstitial radiation therapy for clinically localized prostate cancer. JAMA.

[24] Zelefsky MJ, Yamada Y, Pei X, Hunt M, Cohen G, Zhang Z (2011). Comparison of tumor control and toxicity outcomes of high-dose intensity-modulated radiotherapy and brachytherapy for patients with favorable risk prostate cancer. Urology.

[25] Merrick GS, Wallner KE, Butler WM (2004). Patient selection for prostate brachytherapy: more myth than fact. Oncology (Williston Park).

[26] Blasko JC, Wallner K, Grimm PD, Ragde H (1995). Prostate specific antigen based disease control following ultrasound guided 125iodine implantation for stage T1/T2 prostatic carcinoma. J Urol..

[27] Stokes SH, Real JD, Adams PW, Clements JC, Wuertzer S, Kan W (1997). Transperineal ultrasound-guided radioactive seed implantation for organ-confined carcinoma of the prostate. Int J Radiat Oncol Biol Phys.

[28] Stone NN, Stock RG (2013). Prostate brachytherapy in men with gland volume of 100cc or greater: Technique, cancer control, and morbidity. Brachytherapy.

[29] Stone NN, Stock RG (2007). Long-term urinary, sexual, and rectal morbidity in patients treated with iodine-125 prostate brachytherapy followed up for a minimum of 5 years. Urology.

[30] Chen AB, D’Amico AV, Neville BA, Earle CC (2006). Patient and treatment factors associated with complications after prostate brachytherapy. J Clin Oncol.

[31] Pinkawa M, Fischedick K, Piroth MD, Gagel B, Borchers H, Jakse G (2007). Prostate-specific antigen kinetics after brachytherapy or external beam radiotherapy and neoadjuvant hormonal therapy. Urology.

[32] Mahmood U, Pugh T, Frank S, Levy L, Walker G, Haque W (2014). Declining use of brachytherapy for the treatment of prostate cancer. Brachytherapy.

[33] Instituto de Pesquisas Energeticas e Nucleares-IPEN-CNEN/SP Brazilian demand for iodine-125 seeds in cancer treatment after a decade of medical procedures.

